# An embodied and ecological approach to skill acquisition in racecar driving

**DOI:** 10.3389/fspor.2023.1095639

**Published:** 2023-02-22

**Authors:** Gal Ziv

**Affiliations:** Motor Behavior Laboratory, the Levinsky-Wingate Academic Center, Wingate Campus, Netanya, Israel

**Keywords:** racecar driving, ecological psychology, embodied cognition, motor learning, affordances

## Abstract

Racecar driving is a fast-paced sport that presents the driver-athlete with many perception-action coupling and decision-making challenges. One question that arises is how racecar drivers deal with the influx of perceptual information and manage to perform successfully in such high speeds and, as a result, very limited time to make decisions and act upon them. In this perspective paper, I suggest that the ecological approach is one theoretical framework that can help researchers understand how skill is acquired in racecar driving. I also suggest that an embodied perception of affordances can provide a good basis for research in the field. Specifically, it is an extended embodied cognition that includes not only the driver's mind and body, but the car itself. In a sense, the driver and the car are embodied into one unit and any perception of affordances should be based on this unit. This paper will also discuss the constraints during a race, the affordances the race driver must perceive and how they change over the course of a race, and how researchers can use a racecar driving paradigm to study human perception and action from an embodied and an ecological approach. Specifically, because the driver is seated, measuring EEG and eye movements is relatively simple and can provide additional information on drivers' visual perception of affordances, and their ability to act upon them.

## 1 Introduction

Racecar driving is an inherently fast-paced and dynamic sport that requires drivers to make decision and act upon them under extreme time constraints. Driver-athletes are required to remain attentive for long durations under many physiological stressors that include, for example, high gravitational (g) forces, heat stress, and cardiovascular stress (1). Under such prolonged physiological stress, drivers must perform complex perceptual-motor tasks required to successfully maneuver the racecar around the track as fast as possible. Understanding how racecar drivers can perform under such conditions is a challenge and the literature on racecar driving is relatively limited. As racecar driving becomes more popular (e.g., TV audience for Formula One races in 2021 was 1.55 billion – a 4% increase from 2020, and 108.7 million for the last race of the season – a 29% increase from the same race in 2020 (2)), it may prove useful to study how racing skills are acquired as more novice drivers will potentially join motorsports in the near future.

I suggest that an ecological perspective (3) can guide research on skill acquisition and performance in racecar driving. From this perspective the individual (in our case a race driver) cannot be separated from the environment (4), and thus research should focus on the individual-environment (driver-racetrack) system. A key concept of the ecological approach is that of affordances – opportunities for action based on the interaction between the animal and the environment (3). For example, for an adult human a chair may afford sitting – that is, the functional fit between the person and the chair creates the affordance of sit-ability. The perception of such affordances underlies the concept of affordance-based control (5). Affordance-based control suggests that the main purpose of perception is to allow us to see the world in terms of what we can do in it, and that successful performance relies on our ability to perceive possibilities for action (5). Indeed, from an ecological perspective, the environment is directly perceived in terms of affordances that provide different opportunities or invitations for actions that guide behavior (6). In this respect, skill learning is seen as the establishment of an adaptive, functional fit between an individual and its environment (7). Based on these key concepts of the ecological perspective, racecar drivers cannot be studied outside the racecar and the environment of the racetrack. This implies that we should study performance in real-world scenarios or design tasks and environments that simulate these scenarios, which aligns with Brunswik's (8) idea of representative design for experiments (see also (9), for sport-related representative task design).

Moreover, racecar drivers' performance will be affected by their ability to perceive relevant affordances such as the possibility or invitation to overtake another car (i.e., overtake-ability affordance), and the potential to drive through a turn at the highest speed possible (i.e., turn-ability affordance). One can also argue that such affordances are related to the constraints imposed on the driver. First characterized by Newell (10), constraints can be divided into three categories: (1) Organismic/Individual constraints (e.g., height, reaction time, motivation); (2) Task constraints (e.g., goal of task, rules of competition); and (3) Environmental constraints (e.g., visibility, outside temperature). Multiple constraints from these three categories interact over varying timescales, influencing the development of skill (11).

In addition, I argue that the driver's perception-action coupling cannot be separated from the car itself, and thus the organism of interest is the driver-car unit. This statement relates to the concept of embodied cognition which suggests that we should study the mind in its relationship to the organism's physical body that interacts with the world (12), or in race driving, understand the mind in its relationship to a driver-racecar unit. More recently, Raab and Araújo (13) suggested that to understand the relationship between individuals and their environment, an embodied cognition perspective is required. The embodied cognition perspective is influenced, at least in part, by the works of late 19th and early to mid-20th century philosophers such as John Dewey (see for example (14)) and Maurice Merleau-Ponty (15). Dewey (14) suggested that “*Every “mind’ that we are empirically acquainted with is found in connection with some organized body. Every such body exists in a natural medium to which it sustains some adaptive connection…”* (*p*.277). Merleau-Ponty (15) wrote that: “*…in so far as my body is, not a collection of adjacent organs, but a synergic system, all the functions of which are exercised and linked together in the general action of being in the world…”* (*p*. 272). Embodied cognition suggests that the body contributes to cognition and influences our perception, and that performance emerges from coupling of the nervous system, the body, and the environment (16). In this respect, Mace (17) suggested that to understand perception we should “Ask not what's inside your head, but what your head's inside of” (*p*. 43). This concept contrasts with the more standard cognitive approach suggesting that our brain is locked inside our head, separated from the environment, and receives impoverished environmental information. Based on this poor sensory input and internal mental representations of the world, the brain is required to provide behavioral solutions based on its best guess of what is currently required (18).

It is important to note that several studies have examined the ecological approach to driving (e.g., (19–21)) and one may ask why racecar driving would be any different as it may appear that race driving is *just like regular driving but faster*. I argue that there are fundamental differences between regular, passenger car driving and racecar driving. Indeed, Lappi and Dove (22) suggested that when seeing elite athletes perform, we know that we are not capable of doing what they do. However, our feeling on racecar diving may be different. Lappi and Dove (22) add that: “*…for many people the brain will go Well, you know, I could probably do that – they”re just sitting there and turning the wheel, what*”*s the big deal? When the casual observer looks at a racing car at speed, the natural thing is to relate it to your everyday experience: More of the same, just at a higher level of speed (and risk), the brain says. The tiny cues that tell how the vehicle is being balanced, how it is manipulated to extract maximum speed, may be too subtle to register to the non-expert eye.”* (*p*. 20–21). Indeed, like in many other sporting activities, racecar driving requires intricate perceptual-motor skills that are mostly unattainable by those who do not practice it. Therefore, despite the available literature on driving passenger cars, examining racecar driving is an endeavor worthy of independent effort.

The ecological perspective and the embodied cognition perspective are both important for the understanding of human performance as cognition is both embodied and embedded (in the environment) (23). Therefore, the purpose of the current perspective paper is to show how embodied cognition and an ecological perspective can explain skill acquisition in racecar driving. First, I will briefly discuss the embodied cognition of the racecar driver. Second, I will discuss the main constraints in racecar driving. Third, I will discuss the possible affordances in race driving. Finally, I will provide avenues for future research based on embodiment and on the ecological approach.

## 2 Embodied cognition in racecar driving

Wilson (12) discussed six views of embodied cognition, some of which are particularly relevant to racecar driving. One view is that cognitive activity is situated in a real-world environment, involves perceiving environmental information, and executing motor activities accordingly (i.e., perception-action coupling). Driving is a situated cognitive activity, made for action (i.e., driving the car successfully), and best understood when examined in real-world scenarios. However, conducting research in a real-world racing scenario maybe difficult and impractical. In this respect, representative, high-fidelity racecar simulators – specifically, simulators with high action fidelity in which performance is similar among the simulator and the simulated system (24) – can be of great benefit for the study of race drivers. Simulators are unlikely to completely simulate the real environment and transfer of learning is a good measure of action fidelity (24). In the case of racecar driving, such action fidelity will mean, for example, that lap time in the simulator may transfer to the real track.

A second view suggests that cognitive activity occurs often under time pressure. This is particularly relevant to the fast-paced sport of race driving. For example, racecar drivers have very little time to decide, based on the continually changing information available (e.g., distance to the optimal braking point before a turn, speed, position of cars on track), whether to overtake another car before they need to start braking as they approach a turn. If drivers miscalculate, they may brake too late and approach the turn too fast. This may lead to wheel lockups^1^ that will cause faster tire degradation, or to completely missing the turn and losing valuable lap time. In the worst-case scenarios, such wrong decisions can lead to crashing into the other car or into the barriers.

A third view of embodied cognition is that the environment can be considered as a part of a cognitive system. Wilson (12) explains that: “*The forces that drive cognitive activity do not reside solely inside the head of the individual, but instead are distributed across the individual and the situation as they interact.”* (*p*. 630). This means that the cognitive system encompass the mind and the physical body, but also objects in the near environment (12). In racecar driving, I suggest that embodied cognition includes, at the very least, the mind of the race driver, his/her physical body, and the racecar itself. This will be important when we try to understand race drivers' constraints and perception of affordances on the racetrack. Such affordances relate to a driver-racecar unit, and not only to the physical body of the driver. In regular driving, Dant (25) discussed in depth the importance of examining the driver-car unit and “The Embodied Driver-car” (*p*. 71). I suggest that, similarly, we should examine in depth the driver-racecar unit.

## 3 Constraints in racecar driving

**Figure 1:**
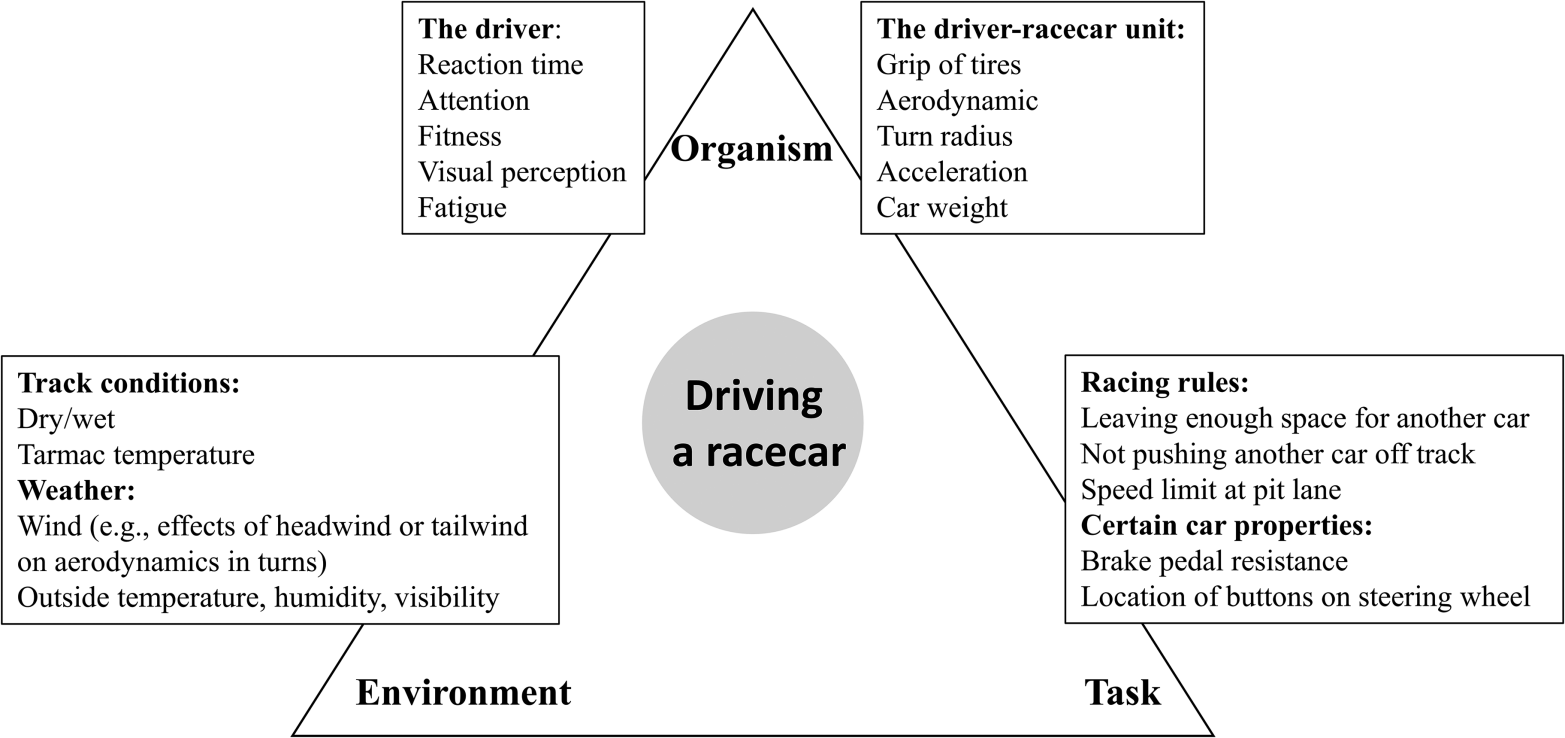
The constraints in racecar driving.

Figure 1 presents some of the constraints racecar drivers encounter based on the Newell's (10) categories. Environmental constraints include outside temperature and humidity, the temperature of the tarmac, wind conditions, etc. Some functional constraints of the organism include reaction time, visual perception, and attentional capabilities. Task constraints include the rules of the race (e.g., how much space a driver must leave for an opponent driver on the tarmac, speed limits in pit lane).

However, when it comes to the properties of the car, we need to make a distinction between properties that present task constraints and properties that cannot be separated from the driver and represent the constraints of the driver-racecar unit (and thus relate to the organism). Newell (10) suggested that machines (such as cars or bicycles) can be considered as task constraints. Such task constraints are related to the way the driver operates the car and can include the responsiveness of the brake pedal and the location of the various functional buttons on the steering wheel. However, other properties of the car cannot be separated from the driver, especially from an embodied approach. Constraints of this driver-racecar unit (organism) include the size of the car, acceleration properties, tire grip, weight, etc. Some of those constraints are fixed (e.g., size of car), and some are dynamic (e.g., weight of car reduced from lap to lap as fuel is being consumed, tire grip reduced from lap to lap as tires degrade). The constraints of this driver-racecar unit are crucial for understanding how drivers perceive affordances and learn to act upon them.

## 4 Affordances in racecar driving

Perhaps the most intuitive affordance that racecar drivers must perceive is overtake-ability. Drivers must be able to recognize a gap and decide whether they have the space (spatial constraint) and the time (temporal constraint) to overtake another car. Several studies have examined drivers' perceptions of affordances (20, 21, 26). However, when driving passenger cars safely according to the rules, there is little or no temporal constraints. Drivers overtake in open roads when there is plenty of time to complete the overtaking maneuver. Racecar drivers face a more complex problem. The driver-racecar unit must pass through a gap in a very limited amount of time. This added temporal constraint creates a great challenge for perceiving the overtake-ability affordance in racecar driving. Indeed, Fajen et al. (27) suggested that in fast-paced dynamic sports affordances appear and disappear in an instant and that to succeed, “*athletes must be acutely aware of the ever-changing opportunities for action afforded by the situation”* (*p*. 80). For the racecar driver, an opportunity to overtake may be available one moment only to disappear in the next one.

Another factor that may affect the perception of overtake-ability is the environment that creates and surrounds the gap the drivers face. Hackney et al. (28), for example, showed that individuals behave differently when passing through an aperture made of inanimate objects or of other individuals. When the obstacles that create an aperture are other individuals (rather than inanimate objects), the critical size of the aperture that causes participants to rotate their shoulders (rather than walk straight through) is larger, possibly to account for individuals' need for personal space from others. This finding shows that it is not only the size of the gap that matters, but that the objects or organisms that create the gap matter as well. We can expand on this finding and ask for example: (1) do novice drivers perceive overtake-ability only when a larger gap is available (e.g., because they are more careful to avoid the objects that create the gap)?, and (2) does the size, distance, material, of the objects that creates the gap between the opponent car and the edge of the track matter? For example, do drivers perceive overtake-ability opportunities more easily when there is space between the edge of the track and the barrier compared with a condition in which the barrier is constructed right on the edge of the track (like in many street circuits).

Another question in this context is how accurately individuals assess the limits of the vehicles they operate. This is relevant for driving passenger cars as well. Schwebel and Yocom (21) (Study 1), reported only modest accuracy in drivers' perception of their car's affordances. In contrast, Morice et al. (20) found that drivers are sensitive to their car's limits and select when to overtake by perceiving an overtake-ability affordance. However, in regular driving conditions, the time constraints when legally overtaking on a straight road are more lenient, and the gaps are relatively large. In racecar driving, the question is how drivers develop the skills to perceive overtake-ability in high speeds, with gap sizes that are many times just enough for the racecar to pass through, and with very limited time before they must brake as they approach an upcoming turn. One possible explanation for the ability of racecar drivers to accomplish such overtaking maneuvers is that they are better at attuning to the relevant environmental information at high speeds. While this may certainly be part of the explanation, racecar driving is fundamentally different than passenger car driving and thus requires specific perceptual-motor capabilities.

In addition, if individuals can learn to accurately assess whether a vehicle they operate pass through a gap, is there a limit to this ability? A car is a relatively small vehicle, but can operators of heavy machinery or passenger airplanes do the same? I suggest that there is a limit for such an embodied machine-operator system. It is unlikely, for example, that a pilot taxiing on a runway can accurately perceive whether the tip of the wing passes through a certain gap. Indeed, airports have strict taxiing rules that prevent the pilot from the necessity to perceive such affordances. So, two relevant questions remain: (1) how racecar drivers learn to accurately assess the driver-racecar unit's overtake-ability under extreme time constraints, and (2) Does the size of the vehicle affect the ability and the time it takes to acquire the skill of accurately assessing the driver-racecar unit's affordances.

Identifying gaps is not the only affordance a racecar driver must perceive. The racing driver needs to maneuver the car as fast as possible around the track and maintain the highest speed possible when engaging a turn, without losing grip. The car's grip and the ability to maintain the highest speed when turning depends on many factors such as the tire type, temperature, and condition, the car's aerodynamic setup, and the weather conditions (i.e., wet tarmac, wind conditions). These variables will decide when drivers break before the turn, and how fast they reach maximum throttle (i.e., how fast can they step on the gas pedal) as they speed out of the turn. Some of those variables are constant (e.g., type of tire until pitting for tire change, tarmac temperature if weather is stable), but other variables change continuously. Specifically, tire grip is gradually reduced as they are degraded from lap to lap and thus the perception of turn-ability must change as well. For example, when engaging a turn, a new set of tires will allow drivers to maintain greater speeds and to be more aggressive on the throttle as they speed out of the turn. After a few laps, these same tires no longer allow these actions. The tires now provide less grip, and drivers must calibrate their perception of turn-ability. Drivers should now accelerate more smoothly, or they will lose grip. This is a great challenge for drivers. On one hand, they want to push the car to the edge of grip to achieve the highest speed as they accelerate from the apex of the turn onto the straight. However, if they push too hard, they risk losing the car. This balancing act, that must be constantly evaluated as the tires degrade from lap to lap requires the driver to be highly attentive throughout the race to the condition of the car and to perceive the correct affordances accordingly.

Finally, racing drivers also need to defend from other drivers who try to overtake them. In these situations, the defending driver can choose where to position the car on the track so the driver trying to overtake will have a harder time doing so. As Withagen (29) suggested, affordances can be seen not only as possibilities or opportunities for action, but also as invitations for actions. In this respect, defending drivers can “*invite”* drivers that try to overtake them to a specific location on the track by, for example, creating a large inviting gap in one side of the track. The side of the track, for example, that would require the overtaking driver to brake earlier, or to accelerate slower, or to miss the apex, and thus fail in their overtaking attempt. To do so, the defending driver should have an intimate knowledge of the situation by perceiving the relative locations and speeds of their car and the car of the other driver, and then realize how to create an “affordance trap” that invites the overtaking driver to a suboptimal overtaking solution.

## 5 Where do we go from here? research in racecar driving

Racecar driving provides an excellent framework for studying skill acquisition and expert performance (See Lappi (30) for a list of 12 features that make it so). From an embodied an ecological perspective, driving simulators can provide high-fidelity representations of the real-world task. These simulators are relatively cheap and can be incorporated in a virtual reality (VR) environment. Specifically, such simulators can provide a model that considers many of the relevant variables that affect drivability. These variables include, among other things, the type of tires, tire temperature and degradation over time, brake temperature, weather conditions, car weight, etc. In addition, the simulator can provide the driver with haptic feedback through the steering wheel. Taking into account the variables that affect the car's grip and the haptic feedback the driver receives should allow for high action fidelity although it remains to be seen whether performance in the simulator transfers well to the racetrack.

However, it is important to note that the physical exertion and physiological stress related to racecar driving cannot be simulated. Specifically, we cannot simulate the high g-forces that drivers encounter while racing. These g-forces can have negative effects on drivers' attention and fatigue. Therefore, simulator studies should be complemented by real-world racecar driving research. Studies on the actual racetrack can help us understand the relationships between gaze behavior, EEG signals related to perception-action coupling, and driving performance under stressful conditions (e.g., heat stress, high g loads) that cannot be simulated. It has been shown that EEG (e.g., (31)) and eye movements (e.g., (32)) can be measured during real-world driving. It remains to be seen whether these technologies can provide reliable data while driving a racecar.

I suggest six (out of possibly many more) directions for future research relating to an embodied and an ecological approach to racecar driving:

### 5.1 Differences between experts and novices in perceiving overtake-ability (and acting upon it)

The ability to perceive overtake-ability on track is a necessity for racecar driving. One question is whether experts perceive overtake-ability when there is a gap not much bigger than the width of a racecar, compared with novices who may perceive overtake-ability only when the gap is larger than the width of a racecar. If so, are experts also able to act upon those perceived affordances successfully?

### 5.2 Underlying mechanism of improved perception of affordances in experts

If experts perceive relevant affordances better than novices, how do they accomplish that? One line of inquiry can include the gaze behavior of drivers. In a review of gaze behavior and expert performance, Brams et al. (33) showed that in sports, compared to novices, experts make more fixations of longer durations to areas of interest in the visual field. In this context, expert racecar drivers possibly make more fixations of longer durations to a possible gap. In contrast, novices may spend less time gazing at the gap, and thus detect less visual information relating to overtake-ability. Similarly, when approaching a turn, do experts spend more time gazing at the apex, and then as they reach the apex, direct their gaze towards the straight that follows the turn? Unfortunately, gaze behavior of racecar drivers or differences in gaze behavior between racing drivers and non-racing drivers can be found in only a handful of studies (e.g., (34, 35)) and to the best of my knowledge, those studies did not study gaze from an ecological perspective.

### 5.3 Can we teach novice drivers to improve their perception of affordances?

Training drivers in visual scanning of a racetrack is one direction for research. But there are other behaviors that can lead to improved performance. For example, the way drivers position their car on the track affects their field of view and their ability to detect actionable information. As Gibson (3) suggested “we must perceive in order to move, but we must also move in order to perceive.” (*p*. 213). Racecar drivers can position their cars on track in a way that increases their chances of visually perceiving relevant affordances. Training studies can be conducted in which novice drivers face various racing and overtaking conditions. In such studies, car position and gaze behavior can be easily recorded.

### 5.4 What is embodied in race car driving?

It remains to be seen what constitutes the embodied cognitive system of the driver. Does the cognitive system only include the mind, the physical body, and the car? Does it go beyond the car? Is there a relationship between such embodiment and perception of turn-ability or overtake-ability? Answering such questions can lead to insights on expert race driving performance.

### 5.5 Do electronic sports (esports) drivers have different perception of embodiment and affordances?

Esports racecar driving competitions are of interest and some online e-races are streamed live. Pedraza-Ramirez et al. (36) suggested that esports psychological research is timely. From an embodied cognition approach, we can ask whether the sense of embodiment of esports race drivers differs from that of drivers on a real racetrack (e.g., does the cognitive system includes the virtual car?). Esports drivers can use a variety of set-ups that range from high-fidelity simulators to relatively basic seat-wheel-pedals systems. Add to the mix the use of VR and you will get a rich environment to examine what constitutes the cognitive system of the driver. From an ecological perspective, it is interesting to examine whether the perception of affordances differ when one does not sit in the actual racecar. Studies on these topics can help improve esports driving performance, but also provide insights into embodied cognition and perception-action coupling in general.

### 5.6 Perceptual attunement and (re)calibration

Perceptual attunement refers to the difference between expert and novices in the information they rely on to perform a task and to the ability to rely on more relevant information (27, 37). Expert racecar drivers rely on visual information (e.g., the apex of the turn), auditory information (e.g., engine sound in different gears), and haptic information (e.g., feedback from steering wheel, bodily sensation caused by g forces, sensations of the car losing grip). Research should examine to which of those (or other) variables experts attune to. Attunement to visual information can be examined with the use of eye trackers, but attunement to haptic information may require qualitative methodologies and “think aloud” protocols. Extending perceptual attunement to action, a process of calibration – scaling action to information (38) – is required. Moreover, in the ever-changing conditions of the car (e.g., tire degradation) and the driver (e.g., cognitive and physical fatigue), recalibration of actions is continuously required. Examining how race drivers are able to constantly rescale their actions to the changing information and still operate their racecar on the edge of its grip to maintain the highest average speed possible will be invaluable to the understanding of their perceptual-cognitive-motor abilities.

## 6 Conclusion

The purpose of this perspective paper was to show how an embodied and an ecological approach can be valuable in racecar driving research. Identifying what exactly is embodied and how racecar drivers identify and act upon affordances on the racetrack can improve our understanding of this fast-paced sport and allow us to provide better training for novice drivers. Moreover, such research could potentially improve drivers' safety as improving perception of affordances can reduce decision-making errors. It is also plausible that such studies can provide valuable information regarding other fast-paced sports as well. Hopefully, this paper will encourage researchers to conduct studies on this topic.

## Data Availability

The original contributions presented in the study are included in the article/Supplementary Material, further inquiries can be directed to the corresponding author.

## Footnotes

^1^Wheel lockup means that the wheel stops rotating while the car is still moving, causing the tire to scrape on the tarmac. This can happen when the race driver brakes too hard and can lead to faster tire degradation – specifically in one spot on the tire.
